# An Incidental Discovery of Multiple Sclerosis in an 18‐Year‐Old Male Following Posttraumatic Brain Imaging

**DOI:** 10.1155/crnm/3906019

**Published:** 2026-11-14

**Authors:** Benjamin Limburg, Emily Wilde, Ty Moody

**Affiliations:** ^1^ University of South Dakota: Sanford School of Medicine Sioux Falls South Dakota USA; ^2^ Department of Internal Medicine University of South Dakota: Sanford School of Medicine Sioux Falls South Dakota USA

## Abstract

Multiple sclerosis (MS) is a chronic, debilitating autoimmune disease for which there is currently no cure. Most commonly, MS presents in white, middle‐aged females. However, the pathogenesis of the condition is incompletely understood. We report a rare case of the incidental discovery of MS in an 18‐year‐old male. In‐depth neurological workup following traumatic brain injury secondary to a motor vehicle accident revealed nonenhancing demyelinating lesions in the brain and spine on MRI. Further workup, including CSF positivity for oligoclonal bands, supported the diagnosis of MS, and the patient promptly began disease‐modifying therapy (DMT) with ocrelizumab. As no current preventive screening strategy is in place for MS in the United States, this patient likely would not have started DMT until many years in the future. This case provides a reason to keep MS on the neurologic differential diagnosis even in demographically atypical patients and inspires further discussion regarding the role of early detection in this disease.

## 1. Introduction

Multiple sclerosis (MS) is an autoimmune disease characterized by chronic inflammation, demyelination, gliosis (plaques or scarring), and neuronal loss in the central nervous system (CNS) white matter [1, 2]. MS is one of the most common neurologic disorders in the world, and its incidence and prevalence are expanding in both developed and developing countries [3]. Prominent risk factors include prior Epstein–Barr virus infection, vitamin D deficiency, and cigarette smoking [4]. Increased prevalence in whites, especially those living at higher altitudes, and a female‐to‐male ratio of 3:1 lend credence to these risk factors but also emphasize that risks and pathophysiology are incompletely understood. Most commonly, MS arises between the ages of 20 and 40, yet as many as 10% of cases appear before the age of 18 [2]. The 2024 revisions to the McDonald criteria incorporate the optic nerve as a fifth region for dissemination in space and allow cerebrospinal fluid–specific oligoclonal bands (OGBs) or kappa free light chains to support diagnosis, while recognizing MRI biomarkers such as the central vein sign and paramagnetic rim lesions [5]. These updates facilitate earlier and more accurate diagnosis, allowing timely initiation of disease‐modifying therapies (DMTs) that can reduce disease activity and delay disability. Although no cure currently exists, advances in treatment have improved long‐term outcomes and quality of life for patients with MS [6, 7].

The following is a rare presentation of MS in an 18‐year‐old male who sought emergency care after his motorized scooter was struck by a car. Among other injuries, he experienced traumatic subdural and subarachnoid hemorrhaging, leading to subsequent follow‐up neuroimaging. These studies incidentally revealed evidence of inactive demyelination of the brain, leading to the diagnosis of MS. This is a case of a radiologically isolated syndrome (RIS), which, according to a cohort study by Okuda et al., will progress to clinically definite MS (CDMS) within 5.4 years [8].

## 2. Case Presentation

An 18‐year‐old white male presented to the local emergency department via EMS after his motorized scooter was hit by a car in early July 2025. Upon arrival, the patient was uncooperative, incoherent, and somewhat combative with a GCS of 9. He was intubated to prevent further injury, and history could not be obtained at the time. The initial CT scan was significant for acute subdural and subarachnoid hemorrhages and a left temporal bone fracture. The patient was then transferred for a higher level of care, including neurosurgical services.

As part of the trauma workup, an MRI of the head and cervical spine was obtained. The brain MRI revealed significant findings, including round and oval‐shaped foci throughout the left cerebrum in the frontal and frontal insular regions, which were intraaxial with susceptibility artifacts and exhibited faint areas of linear T1 intrinsic shortening (Figure 1). The largest lesion measured 3.2 cm. T2/FLAIR hyperintensity diffusely throughout the cortical, subcortical, periventricular, and posterior fossa regions supported concern for demyelination (Figure 2). Cervical spine MRI was significant for nonenhancing, multifocal, scattered, faint foci of T2 intramedullary signal hyperintensity of the cervical cord, including the C2 level, which measured 1.2 cm, and the C4‐C5 level, which measured 1.6 cm. All lesions were nonenhancing and considered incidental findings.

**FIGURE 1:**
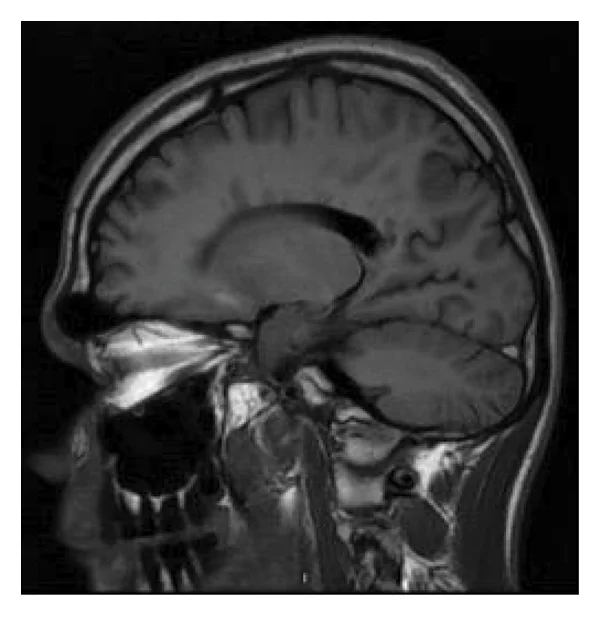
Sagittal T1 brain MRI highlights extensive white matter signal changes, specifically in the periventricular regions, concerning for demyelination.

**FIGURE 2:**
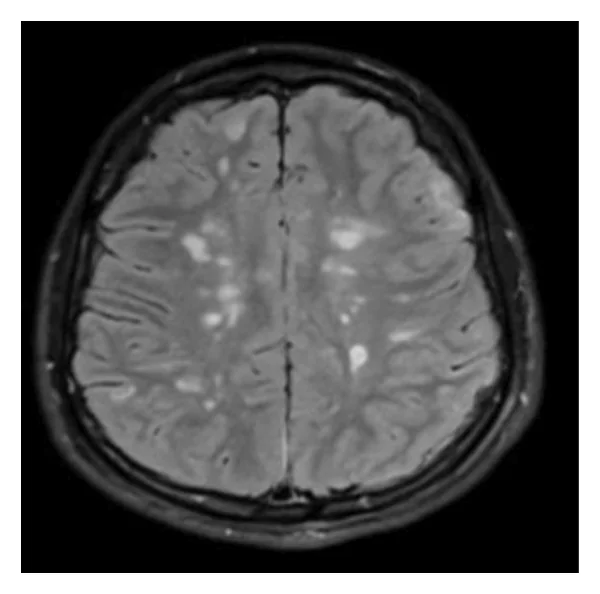
Brain MRI showing diffuse round/oval‐shaped cortical and subcortical foci of T2 FLAIR signal hyperintensity concerning for demyelination with associated vasogenic edema.

A neurology consult was obtained based on the concern for a demyelinating process, specifically MS. Upon obtaining history from family and the patient, it was determined that the patient experienced right upper extremity weakness in mid‐2021. Extensive workup, including EMG, was performed in November of that year and was negative, with a central cause ruled out and the weakness self‐resolving. The patient denied any other history of transient neurological symptoms, including vision changes, vertigo, weakness, or ataxia. Visual changes were difficult to assess given legal blindness in his right eye due to an accident in childhood. Family history was insignificant for neurologic pathologies, including MS. The neurologic physical exam was unremarkable. Further neurologic workup included MRI of the thoracic spine in addition to the previously obtained head and cervical spine images. Thoracic spine MRI was significant for a focus of nonenhancing T2/STIR hyperintensity at the T4 level, with additional small, scattered foci of nonenhancing high signal versus artifact. Along with the intracranial findings, these were thought to represent additional sequelae of demyelinating disease. Since no gadolinium‐enhancing lesions were found, the patient was cleared from a neurologic perspective to be followed as an outpatient after he recovered from his head trauma.

Upon initial evaluation by outpatient neurology, a lumbar puncture was performed in mid‐August 2025 to further investigate a diagnosis of MS. Cerebrospinal fluid analysis was significant for OGBs, and MOG and AQP‐4/NPG antibodies were negative, further supporting the diagnosis of MS.

At a follow‐up visit in early September 2025, treatment options were discussed, and it was decided he would begin ocrelizumab as DMT. No new neurologic symptoms were identified on physical exam at either outpatient visit, and right upper extremity weakness 4 years prior was insignificant. It has been determined that this is a RIS. The patient was seen in clinic 6 months after the decision was made to begin ocrelizumab, and he has been doing well. He denies adverse effects from the two ocrelizumab infusions thus far and denies any new neurological symptoms. The patient and his family wish to continue DMT for RIS to prevent disease progression.

## 3. Discussion

MS is an autoimmune demyelinating disease of the CNS white matter. It is diagnosed most commonly via the McDonald criteria with the support of MRI and CSF analysis. The McDonald criteria state that patients must display evidence of two or more lesions “separated by both time and space.” [6] “Separation in time” refers to episodes lasting more than 24 h, separated by a month or more; “separation in space” designates pathology in two or more noncontiguous CNS white matter tracts. Most commonly, MS occurs in white, middle‐aged females. However, about 10% of cases begin prior to age 18 [7].

The incidental discovery of MS in an 18‐year‐old patient illustrates the duality of any medical investigation, gathering answers to questions for which the test is indicated, along with unintended discoveries. For MS, the workup process often begins after clinically suspicious symptoms are reported. Ultimately, this can yield a bona fide diagnosis of MS or what is known as a clinically isolated syndrome (CIS): neurologic disturbance due to a single white matter lesion which may or may not progress to MS [9]. On the contrary, a RIS describes the scenario in which white matter lesions are incidentally demonstrated on brain or spine MRI in the absence of clinical episode history [10]. The most recent RIS diagnostic criteria are as follows: lesions satisfying at least three of the four McDonald separation‐in‐space criteria, or at least one T2 hyperintense lesion in two or more CNS locations plus at least one of a spinal cord lesion, CSF‐restricted OGBs, or a new enhancing lesion on follow‐up MRI [11]. This patient indeed fulfills the criteria for RIS, given he was asymptomatic at the time of presentation and had numerous T2 brain and spinal cord lesions, along with positive OGBs. The ARISE and TERIS studies both showed significant supporting data for treating RIS, as seen in this young patient, in delaying disease progression and disability [12, 13]. Three clinically distinct variations of MS are described: relapsing MS (RMS), primary progressive MS, and secondary progressive MS [2]. One could assume the MS subtype in this case to be RMS, given his subjective return to neurologic baseline. However, time is needed to better understand the clinical course of this patient’s disease.

MRI is informative in many cases of MS. Yet, especially early in the disease course, establishing “separation in time” can be difficult, along with specifically distinguishing MS from other potential etiologies of neurologic disease [14]. The differential diagnosis in the case of this patient’s demyelinating lesions was initially broad, including acute disseminated encephalomyelitis, Lyme disease, neuromyelitis optica, sarcoidosis, vascular malformations, and vasculitis. Other paraclinical tests, especially CSF analysis, are utilized to rule out such suspected pathologies. In MS, CSF findings commonly include increased IgG, further supported by detection of OGBs, normal or mildly elevated protein, and mononuclear cell pleocytosis [2]. OGBs are positive in more than 90% of patients with MS. This patient’s OGB positivity, along with negative MOG and AQP‐4 antibodies (markers of MOG‐antibody–associated disease and neuromyelitis optica, respectively), provided appropriate confirmation for the final diagnosis of MS.

Unfortunately, there is no cure for MS, and the goal of treatment is to reduce relapses and slow disease progression. DMTs are often grouped along a spectrum which includes low, moderate, moderate to high, and high efficacy [15]. High efficacy agents are natalizumab, alemtuzumab, ocrelizumab, ofatumumab, and rituximab. Ocrelizumab, an anti‐CD20+ B cell agent administered as an intravenous infusion, was chosen for this patient. Variations in DMT strategy exist, and the choice in management is often clinician‐dependent.

This case emphasizes the potential for incidental detection of serious disease inherent with many modern diagnostic tests. The patient presented with a traumatic head injury, and it was only after routine follow‐up for residual brain trauma that his chronic demyelinating lesions were discovered. Subsequent CSF analysis supported the diagnosis, and he was started on DMT without delay. By discovering this patient’s disease early in its course, there is hope his disease burden can be minimized throughout the course of his life. Cases such as this reiterate the necessity of keeping MS on the neurologic differential diagnosis even in patients outside the typical demographic profile. In addition, although no asymptomatic screening strategy currently exists for MS in the United States, as diagnostic and treatment capabilities continue to improve, further discussion should continue as to the role of early detection in this debilitating disease.

## Funding

No financial support or funding from any external sources was utilized in the process of writing this manuscript.

## Consent

Written informed consent was obtained from the patient and his legal guardian for publication of this case report and accompanying images.

## Conflicts of Interest

The authors declare no conflicts of interest.

## Data Availability

The data that support the findings of this study are available on request from the corresponding author. The data are not publicly available due to privacy or ethical restrictions.
